# Relationships between weekly changes in salivary hormonal responses and load measures during the pre-season phase in professional male basketball players

**DOI:** 10.5114/biolsport.2023.114290

**Published:** 2022-06-01

**Authors:** Paulius Kamarauskas, Inga Lukonaitienė, Mindaugas Kvedaras, Tomas Venckūnas, Daniele Conte

**Affiliations:** 1 Institute of Sport Science and Innovations, Lithuanian Sports University, Kaunas, Lithuania

**Keywords:** External load, Internal load, RPE, Salivary markers, Load measures, Salivary cortisol, Salivary testosterone

## Abstract

The aim of this study was to assess the relationships between weekly changes in external and internal load considered separately and jointly and salivary hormonal responses during the pre-season phase in professional male basketball players. Twenty-one professional male basketball players (mean ± standard deviation, age: 26.2 ± 4.9 years; height: 198.7 ± 6.7 cm; body mass: 93.2 ± 10.0 kg) were assessed during 5 weeks of the pre-season phase. External load was measured using microsensors and reported as PlayerLoad (PL) and PL/ min. Internal load was calculated using the session rating of perceived exertion scale (sRPE-load), summated heart rate zones (SHRZ) and percentage of maximal heart rate (%HR_max_). Salivary hormone responses were monitored weekly by measuring testosterone (T), cortisol (C), and their ratio (T:C). The relationships between weekly changes in load measures considered separately and jointly and hormonal responses were assessed using linear mixed model analysis. No significant (p > 0.05) relationships were evident between weekly changes in T, C or T:C with external and internal load measures considered separately (R^2^-conditional = < 0.001–0.027) or jointly (R^2^-conditional = 0.028–0.075). Factors other than measured loads might be responsible for weekly changes in hormonal responses and therefore external and internal load measures cannot be used to anticipate weekly hormonal responses during the pre-season phase in professional basketball players.

## INTRODUCTION

In basketball, the pre-season is a crucial phase during which coaches aim to prescribe optimal loads and a sufficient amount of sportspecific training for their players to obtain the best possible physical condition for the upcoming in-season phase [1–3]. The implementation of the aforementioned goals necessitates having precise control of the training and recovery process to avoid inappropriate loading that may lead to an insufficient physiological or psychological response, too high stress, increased risk of injuries, illnesses, overtraining or overreaching [4, 5]. Therefore, the prescription of appropriate loads and recovery interventions seems to be a key factor of the pre-season phase, thus emphasizing the importance of systematically monitoring the load sustained by the basketball players.

Load monitoring in basketball can be categorized as measures of external and internal load [5, 6]. External load is described as the imposed physical load, stimuli or dose, while internal load is considered as the physiological, psychological or perceptive response [5, 6]. Previously, the dose-response relationship between load measures during the pre-season and the in-season phases in semi-professional male basketball players has been investigated [6]. The findings revealed strong (r = 0.53–0.69) to very strong (r = 0.74–0.88) relationships between external (i.e. PlayerLoad [PL]) and internal (i.e. summated-heart-rate-zones model [SHRZ]; session rating of perceived exertion load [sRPE-load]) load measures, with stronger relationships occurring during training sessions (r = 0.69–0.88) than basketball matches (r = 0.53–0.69) [6]. These findings suggest a better anticipation of internal loads in response to the prescribed external loads and can enhance the possibility to organize properly the training and recovery process.

In addition, the analysis of hormonal responses in basketball has been suggested as fundamental to determine the level of stress and recovery, and in monitoring the risk of overtraining and non-functional overreaching [7]. Specifically, testosterone (T), cortisol (C), and their ratio (T:C) were indicated as useful markers to determine the balance between anabolic and catabolic processes [8], allowing a better understanding of training and recovery processes in basketball to be attained. Increased levels of T were reported to show an appropriate recovery, while decreased levels of T and increased levels of C indicate possible risk of overtraining, non-functional overreaching and reduced performance [9]. Consequently, the comprehension of the influence of changes in load measures on changes in hormonal responses would help in the process of monitoring basketball players and designing and implementing sound training sessions during the pre-season phase.

Despite the relevance of this topic, only one study has analysed the relationships between changes in hormonal responses and changes in external and internal load measures in semi-professional, male basketball players during the in-season phase [10], with non-significant trivial-to-moderate (p > 0.05; r = -0.01–0.36) relationships found [10]. Nevertheless, the applicability of these results for professional male basketball players during the pre-season phase is questionable. Indeed, it was previously reported that professional male basketball players usually experience higher loads than do semiprofessional male players [1]. Moreover, it was reported that the preseason phase in male basketball is characterized by higher loads compared to the in-season phase [2]. Thus, in order to determine whether load measures can be used for anticipation of changes in hormone levels in other basketball populations during different phases of the season and specifically during the pre-season phase in professional male basketball players, it is important to assess whether weekly changes in hormonal responses are influenced by weekly changes in external and internal load measures. Therefore, the aim of this study was to assess relationships between weekly changes in external and internal load measures and salivary hormonal responses during the pre-season phase in professional male basketball players.

## MATERIALS AND METHODS

### Subjects

Twenty-five professional male basketball players from two teams competing in European level leagues (Team 1: FIBA Basketball Champions League, n = 12; Team 2: EuroCup Basketball League, n = 13) were recruited for the initial study sample. Due to attendance of training sessions being lower than 75%, four players (Team 1: n = 2; Team 2: n = 2) were excluded from the analysis and the final study sample consisted of 21 players (mean ± standard deviation [SD], age: 26.2 ± 4.9 years; height: 198.7 ± 6.7 cm; body mass: 93.2 ± 10.0 kg). All investigated players were over 18 years of age and were familiarized with the aims, procedures, requirements, possible risks, and benefits of the study, providing written consent before participating. Ethical approval was obtained from the Kaunas Regional Research Ethics Committee review board (No. BE-2-97).

### Design

An observational study took place during 5 weeks of the pre-season phase for Team 1 (2019–2020 FIBA Basketball Champions League; August 16 to September 20, 2019) and Team 2 (2020–2021 EuroCup Basketball League; August 10 to September 13, 2020). Players were familiarized with all study procedures two weeks before the start of data collection. During the monitoring period, internal and external load measures were assessed across 45 training sessions and 6 pre-season matches (5 wins by 20.8 ± 16.6 points; 1 tie match) for Team 1 and 37 training sessions and 5 pre-season matches (3 wins by 35.3 ± 18.2 points; 2 losses by 13.0 ± 15.6 points) for Team 2. An experimental day for saliva collection was selected individually for both teams, considering their day 1 of the pre-season phase. Saliva samples were collected during an experimental day at the beginning of each week of the pre-season phase, and specifically, hormonal responses for Team 1 were monitored every Friday, and for Team 2 every Monday.

### Procedures

Salivary analysis were conducted to measure weekly changes in T, C and T:C. To avoid any possible variation due to the circadian rhythm, saliva samples were collected at the same time of the day (17:00) [11]. Players were instructed not to eat, brush their teeth, or consume any drinks other than still water during the 90 min prior to saliva collection [12]. Before collection, players rinsed their mouth with distilled water [13], remained seated for 30 s, then spat all saliva from their mouth. Afterwards, players waited in a seated position for ~10 min before starting collection of the saliva sample by spitting into 15-ml ultrapure polypropylene SaliCap tubes through a polypropylene straw (IBL International, Germany) [14]. Collected saliva samples were stored at –20°C for subsequent analysis. T and C were determined in duplicate using an enzyme-linked immunoassay (IBL International, Germany) following the manufacturer’s instructions. The intra-assay coefficient of variation for T and C was 2.35% and 3.14% for Team 1, and 2.51% and 3.22% for Team 2, respectively.

To assess external load during training sessions and matches, PlayerLoad (PL) and PlayerLoad per minute (PL/min) were measured using triaxial accelerometers ClearSky T6 (Catapult Innovations, Melbourne, Australia) sampling at 100 Hz to calculate instantaneous movement demands (in arbitrary units, AU) [6]. PL is a modified vector magnitude determined as the square root of the sum of the squared instantaneous rate of change in acceleration across three motion planes [6]. Data were processed using OpenField software (version 1.18, Catapult Innovations, Melbourne, Australia) and downloaded for statistical analysis.

The session rating of perceived exertion method (sRPE) was used for the assessment of internal load measures as previously used in basketball [15–17]. Approximately 30 min after each investigated training session or pre-season match, players were asked to rate their sRPE in the absence of peers using the modified Borg 10-point RPE scale (CR-10) [18] via cloud-based software (Google Docs, Microsoft, California, USA). Participants’ compliance was 93% for both Team 1 and Team 2. Players’ sRPE values and the total duration (min) of each investigated training session or pre-season match (including warm-up, breaks, and stoppage time in play) were used to calculate sRPE load (sRPE-load), using the following formula: sRPE-load = sRPE × total duration (min) [18].

Internal load was monitored using heart rate (HR) collected using Polar HR sensors and straps (Polar H10, Polar Electro, Kempele, Finland) at a sampling rate of 1 s, and with data downloaded and processed using OpenField software. The summated-heart-rate-zones model (SHRZ) was then calculated as: SHRZ (AU) = (duration in zone 1 × 1) + (duration in zone 2 × 2) + (duration in zone 3 × 3) + (duration in zone 4 × 4) + (duration in zone 5 × 5), where zone 1 = 50%–59.9% of maximum HR (HR_max_), Zone 2 = 60%–69.9% HR_max_, Zone 3 = 70%–79.9% HR_max_, Zone 4 = 80–89.9% HR_max_, Zone 5 = 90–100% HR_max_, and duration is time in min) [19]. The average %HR_max_ response was calculated for each training session and match. The HR_max_ was determined as the peak HR response during a 30–15 Intermittent Fitness Test performed prior to the monitoring period on a basketball court and updated using the peak response evident during training or matches if it exceeded that achieved during the testing session [20].

### Statistical analysis

Mean and SD were calculated as descriptive statistics. Linear mixed model (LMM) analysis, which has been previously suggested to be suitable to assess the relationship across load measures in team sports [17, 21] was used to assess how weekly changes in different external and internal load measures affect separately (i.e. load measures considered singularly) and jointly (i.e. combining the internal and external load measures) weekly changes in hormonal responses. Weekly changes in T, C and T:C were used as dependent variables, while weekly changes in load measures indicating training volume (PL, sRPE-load and SHRZ) and weekly averaged values of load measures indicating training intensities (PL/min, %HR_max_) were used as fixed effects. Player was then used as a random effect with a random intercept and fixed slope. An alpha level of p < 0.05 was set *a priori* for statistical significance. All data were analysed using Jamovi software (version 1.2.27, 2020).

## RESULTS

Descriptive statistics of weekly external and internal load measures and hormonal responses are presented in Table 1. The results of the effect of separate weekly changes in external and internal load measures on weekly changes in hormonal responses are presented in Table 2. The LMM analysis showed no significant effect of weekly changes in load measures on changes in hormonal levels.

**TABLE 1:** Descriptive statistics of weekly load measures and hormone concentration.

Dependent variables	Week 1	Week 2	Week 3	Week 4	Week 5
**Workload variables**					
PlayerLoad^TM^ (AU)	3495 ± 1009	3915 ± 782	3555 ± 706	3469 ± 835	3442 ± 780
PlayerLoad^TM^/min (AU)	3.8 ± 0.6	4.3 ± 0.7	4.6 ± 0.6	4.0 ± 1.1	4.3 ± 0.7
sRPE-load (AU)	4991 ± 1913	5125 ± 811	4870 ± 966	5597 ± 1392	4875 ± 1306
SHRZ (AU)	1412 ± 528	1470 ± 405	1387 ± 303	1415 ± 426	1292 ± 323
%HR_max_ (%)	61.9 ± 3.9	61.5 ± 3.1	62.4 ± 4.0	60.9 ± 3.8	61.2 ± 3.0
**Hormonal responses**					
Testosterone (nmol/l)	0.25 ± 0.12	0.32 ± 0.14	0.28 ± 0.17	0.38 ± 0.17	0.39 ± 0.16
Cortisol (nmol/l)	3.49 ± 1.98	3.12 ± 1.54	2.71 ± 1.43	2.90 ± 1.48	2.58 ± 1.03
Testosterone:Cortisol	0.09 ± 0.06	0.14 ± 0.09	0.12 ± 0.08	0.15 ± 0.08	0.17 ± 0.12

*Notes.* Data presented as mean ± SD. sRPE-load – training load from session rating of perceived exertion scale; SHRZ – summated heart rate zones; %HR_max_ – average percentage value from attained maximum heart rate.

**TABLE 2:** The effect of separate weekly changes in external and internal load measures on changes in weekly salivary Testosterone, Cortisol and Testosterone:Cortisol levels.

Dependent variables	AIC	R-squared Conditional	Fixed effects	Estimate (95% CI)	SE	p-value
Testosterone	-70.000	7.86e^-4^	PlayerLoad^TM^	-4.81e^-6^ (-4.17e^-5^, 3.21e^-5^)	188e^-5^	0.799
	-70.096	0.002	PlayerLoad^TM^/min	-0.008 (-0.049, 0.032)	0.021	0.679
	-70.812	0.011	sRPE-TL	-9.76e^-6^ (-3.00e^-5^, 1.05e^-5^)	1.03e^-5^	0.347
	-70.011	0.001	%HR_max_	-0.001 (-0.011, 0.010)	0.005	0.768
	-70.067	0.002	SHRZ	-1.54e^-5^ (-9.49e^-5^, 6.42e^-5^)	4.06e^-5^	0.706

Cortisol	341.138	0.020	PlayerLoad^TM^	2.89e^-4^ (-1.49e^-4^, 7.27e^-4^)	2.24e^-4^	0.199
	342.329	0.006	PlayerLoad^TM^/min	0.168 (-0.313, 0.649)	0.245	0.495
	340.816	0.024	sRPE-TL	1.75e^-4^ (-6.72e^-5^, 4.17e^-4^)	1.23e^-4^	0.161
	342.529	0.003	%HR_max_	-0.029 (-0.142, 0.083)	0.057	0.606
	342.175	0.004	SHRZ	2.65e^-4^ (-6.88e^-4^, 0.001)	4.86e^-4^	0.587

Testosterone: Cortisol	-148.761	0.011	PlayerLoad^TM^	-1.13e^-5^ (-3.42e^-5^, 1.16e^-5^)	1.17e^-5^	0.337
	-149.921	0.025	PlayerLoad^TM^/min	-0.018 (-0.043, 0.006)	0.013	0.150
	-150.035	0.027	sRPE-TL	-9.59e^-6^ (-2.22e^-5^, 2.99e^-6^)	6.42e^-6^	0.139
	-147.931	0.001	%HR_max_	-9.36e^-4^ (-0.007, 0.005)	0.003	0.755
	-147.928	0.001	SHRZ	-7.85e^-6^ (-5.75e^-5^, 4.18e^-5^)	2.53e^-5^	0.757

*Note.* AIC – Akaike information criterion; CI – confidence interval; SE – standard error. *Abbreviations*. %HR_max_ – percentage of maximum heart rate; SHRZ – summated-heart-rate-zones; sRPE-load – session rating of perceived exertion load.

The effect of weekly changes in joint load measures on weekly changes in hormonal responses are presented in Table 3. The LMM analysis showed that weekly changes in T, C and T:C responses are not affected by joint weekly changes in PL, PL/min, sRPE-load, %HR_max_ and SHRZ (p > 0.05).

**TABLE 3:** The effect of joint weekly changes in external and internal load measures on changes in salivary hormonal responses

Dependent variables	AIC	R-squared Conditional	Fixed effects	Estimate (95% CI)	SE	p-value
Testosterone	-64.268	0.028	PlayerLoad™	3.54e^-5^ (-5.523-5, 1.26e^-4^)	4.62e^-5^	0.446
			PlayerLoad^TM^/min	-0.023 (-0.098, 0.052)	0.038	0.550
			sRPE-TL	-2.42e^-5^ (-5.83e^-5^, 9.91e^-6^)	1.74e^-5^	0.168
			%HR_max_	-5.83e^-4^ (-0.021, 0.020)	0.011	0.956
			SHRZ	-1.09e^-5^ (-2.09e^-4^, 1.87e^-4^)	1.01e^-4^	0.915

Cortisol	347.504	0.039	PlayerLoad™	-1.84e^-4^ (-0.001, 8.91e^-4^)	5.49e^-4^	0.738
			PlayerLoad^TM^/min	0.432 (-0.462, 1.325)	0.456	0.346
			sRPE-TL	1.67e^-4^ (-2.39e^-4^, 5.74e^-4^)	2.07e^-4^	0.422
			%HR_max_	-0.080 (-0.324, 0.164)	0.124	0.521
			SHRZ	4.27e^-4^ (-0.002, 0.003)	0.001	0.723

Testosterone: Cortisol	-146.237	0.075	PlayerLoad™	1.77e^-5^ (-3.73e^-5^, 7.26e^-5^)	2.80e^-5^	0.530
			PlayerLoad^TM^/min	-0.027 (-0.072, 0.019)	0.023	0.255
			sRPE-TL	-2.08e^-5^ (-4.15e^-5^, -2.29e^-9^)	1.06e^-5^	0.053
			%HR_max_	-7.20e^-4^ (-0.013, 0.012)	0.006	0.910
			SHRZ	1.38e^-5^ (-1.07e^-4^, 1.34e^-4^)	6.14e^-5^	0.823

*Note.* AIC – Akaike information criterion; CI – confidence interval; SE – standard error. *Abbreviations*. %HR_max_ – percentage of maximum heart rate; SHRZ – summated-heart-rate-zones; sRPE-load – session rating of perceived exertion load.

## DISCUSSION

This is the first study assessing the effect of separate and joint weekly changes in external (PL, PL/min) and internal (sRPE-load, %HR_max_, SHRZ) load measures on weekly changes in salivary hormone responses (T, C, T:C) during the pre-season phase in professional male basketball players. The LMM analysis revealed that weekly changes in T, C, and T:C responses are not affected by weekly changes in load measures considered separately or jointly.

The use of salivary markers as a tool to analyse the balance between anabolic and catabolic processes in different basketball training typologies has grown during the last decade [8, 11, 22–26]. High levels of T were characterized as an indicator for optimal recovery, whereas decreased levels of T and increased C concentrations were indicated as markers of overtraining and reduced performance [9]. Understanding whether load measures might play a role as potential predictors of hormonal responses might be important in order to help in the process of monitoring weekly load and consequently planning basketball training sessions. To the best of our knowledge, no previous investigation has assessed whether weekly changes in load measures influence changes in hormonal responses during the pre-season phase in professional male basketball players. Our findings revealed that separate weekly changes in external (PL, PL/min) and internal (sRPE-load, %HR_max_, SHRZ) load measures have no influence on changes in salivary levels of T, C and T:C. These results are in line with a previous investigation on semiprofessional male basketball players monitored during the in-season phase, in which trivial-to-moderate relationships were found between changes in levels of T, C, T:C and various external and internal load measures [10]. Interestingly, these similar outcomes indicate that during both in-season and pre-season phases and for both levels of competition (i.e. semi-professional and professional players), changes in hormone concentrations and load measures possess a low commonality in basketball. It should be noted that our results are also in line with other studies investigating different sports [27–29]. Indeed, moderate (p = 0.025; r = 0.551) relationships between internal load (sRPE) and salivary C were found in high-level male and female middle and long-distance runners [27], and non-significant (p > 0.05) associations between internal load (sRPE) and weekly C levels were found in elite male Rugby Union players [28]. Moreover, one previous investigation documented no relationships between weekly measures of external and internal load variables and T and C levels in professional football players during the competitive season [29]. Overall, the results demonstrated that factors other than weekly changes in external and internal load measures considered separately are responsible for weekly changes in hormonal responses in team and individual sports athletes.

The second part of our study was designed to investigate whether weekly changes in load measures considered jointly might have an influence on weekly changes in hormonal responses. In fact, basketball players during training periods undergo different combinations of external and internal load measures that should be taken into account during the monitoring process [30]. Therefore, the use of several measures combined can be expected to provide a clearer picture about players’ loads during specific training periods, and potentially might affect the hormonal responses. Nevertheless, similarly to the separate responses, no influence of joint weekly changes in external (PL, PL/min) and internal (sRPE-load, %HR_max_, SHRZ) load measures on weekly changes in T, C and T:C concentrations was found. Therefore, these findings imply that the investigated physical, physiological, and perceptive load measures have no influence on hormonal responses, suggesting that other measures such as psychological factors should be taken into consideration. Indeed, Moreira et al. [25] found an increase in C levels, despite a significant decrease in perceived exertion load (sRPE-load), during a 4-week in-season phase training period in professional male basketball players, suggesting that salivary hormone concentrations increased due to the high psychological stress of the upcoming playoff phase. Moreover, two review articles indicated that both acute and chronic hormonal responses to training are influenced by physiological (e.g. heart rate, oxygen consumption) and psychological (e.g. self-confidence, pressure) factors [5, 31]. Physiological, psychological, and behavioural stressors of sports training were indicated as the main factors activating or inhibiting the release of T and C [31]. Therefore, a possible explanation of our findings might be that changes in T, C and T:C levels are influenced by a combination of factors other than commonly used external and internal load measures, indicating that measures of hormonal responses might provide an additional insight about players’ responses to the pre-season phase training. Overall, separately or jointly considered factors influencing changes in T, C and T:C levels remain unclear, requiring more investigations to reveal factors having an impact on changes in salivary hormonal levels during training periods in basketball.

Our investigation provides innovative and useful findings for basketball coaches and sports scientists, but there are some limiting factors that should be considered. Firstly, the investigated players participated in data collection after the off-season, in which their load was not monitored, making it difficult to compare the initial fitness level and adaptation progress in relation to its capacity between players, which might influence different internal and hormonal responses during the pre-season phase. Secondly, only external (PL, PL/min) and internal (sRPE-load, %HR_max_, SHRZ) load measures were included in the analysis, while other physiological and psychological factors, or changes in players’ well-being, might be influencing changes in hormonal responses. Therefore, further research to analyse the effect of separate and joint weekly changes in other measures on changes in hormonal responses in professional male basketball players is required.

The findings of the current investigation provide some interesting practical recommendations. Firstly, basketball coaches and scientists should be aware that weekly changes in hormonal responses during the pre-season phase in professional male basketball players are independent from the separate and joint weekly changes in external and internal load measures. Therefore, changes in hormonal responses cannot be anticipated by load measures, and to have a full picture of players’ responses to load, salivary analysis should be included in the process of monitoring basketball players as a separate monitoring tool.

## CONCLUSIONS

The current investigation did not detect a significant relationship between separate and joint weekly changes in external and internal load measures and weekly changes in T, C and T:C during the preseason phase in professional male basketball players, suggesting that other factors or a combination of them might affect hormonal responses. These results suggest that external and internal load measures cannot be used to anticipate weekly hormonal responses during the pre-season phase in professional male basketball players.

## References

[1] Ferioli D, Bosio A, Bilsborough JC, Torre A La, Tornaghi M, Rampinini E. The preparation period in basketball: Training load and neuromuscular adaptations. Int J Sports Physiol Perform. 2018; 13(8):991–9. 29345555 10.1123/ijspp.2017-0434

[2] Aoki MS, Ronda LT, Marcelino PR, Drago G, Carling C, Bradley PS, Moreira A. Monitoring training loads in professional basketball players engaged in a periodized training program. J Strength Cond Res. 2017; 31(2):348–58. 27243913 10.1519/JSC.0000000000001507

[3] Ferioli D, Bosio A, Torre ALA, Carlomagno D, Connolly DR, Rampinini E. Different training loads partially influence physiological responses to the preparation period in basketball. J Strength Cond Res. 2018 Mar; 32(3):790–7. 28146032 10.1519/JSC.0000000000001823

[4] Drew MK, Finch CF. The relationship between training load and injury, illness and soreness: A Systematic and Literature Review. Sport Med. 2016; 46(6):861–83. 10.1007/s40279-015-0459-8 26822969

[5] Impellizzeri FM, Marcora SM, Coutts AJ. Internal and External Training Load: 15 Years On. Int J Sports Physiol Perform. 2019; 14(2):270–3. 30614348 10.1123/ijspp.2018-0935

[6] Fox JL, O’Grady CJ, Scanlan AT, O’Grady CJ, Scanlan AT. The relationships between external and internal workloads during basketball training and games. Int J Sports Physiol Perform. 2020; 15(8):1081–6. 32814307 10.1123/ijspp.2019-0722

[7] Schelling X, Calleja-González J, Torres-Ronda L, Terrados N. Using testosterone and cortisol as biomarker for training individualization in elite basketball: A 4-year follow-up study. J Strength Cond Res. 2015;29(2):368–78. 25144130 10.1519/JSC.0000000000000642

[8] Kamarauskas P, Conte D. Changes in salivary markers during basketball long-term and short-term training periods: a systematic review. Biol Sport. 2022;39(3):673–93. 35959340 10.5114/biolsport.2022.107018 PMC9331333

[9] Coutts A, Reaburn P, Piva T, Murphy A. Changes in Selected Biochemical, Muscular Strength, Power, and Endurance Measures during Deliberate Overreaching and Tapering in Rugby League Players. Int J Sports Med. 2007; 28(2):116–24. 16835824 10.1055/s-2006-924145

[10] Kamarauskas P, Lukonaitienė I, Scanlan AT, Ferioli D, Paulauskas H, Conte D. Weekly Fluctuations in Salivary Hormone Responses and Their Relationships With Load and Well-Being in Semiprofessional, Male Basketball Players During a Congested In-Season Phase. Int J Sports Physiol Perform. 2021; 1–7. 10.1123/ijspp.2021-0106 34686618

[11] Nunes JA, Moreira A, Crewther BT, Nosaka K, Viveiros L, Aoki MS. Monitoring training load, recovery-stress state, immune-endocrine responses, and physical performance in elite female basketball players during a periodized training program. J Strength Cond Res. 2014; 28(10):2973–80. 24736768 10.1519/JSC.0000000000000499

[12] Arruda AFS, Aoki MS, Paludo AC, Drago G, Moreira A. Competition stage influences perceived performance but does not affect rating of perceived exertion and salivary neuro-endocrine-immune markers in elite young basketball players. Physiol Behav. 2018; 188:151–6. 29425971 10.1016/j.physbeh.2018.02.009

[13] Andre M, Fry A, Luebbers P, Hudy A, Dietz P, Cain G. Weekly Salivary Biomarkers across a Season for Elite Men Collegiate Basketball Players. Int J Exerc Sci. 2018; 11(6):439–51.

[14] He CS, Tsai ML, Ko MH, Chang CK, Fang SH. Relationships among salivary immunoglobulin A, lactoferrin and cortisol in basketball players during a basketball season. Eur J Appl Physiol. 2010; 110(5):989–95. 20668874 10.1007/s00421-010-1574-8

[15] Lukonaitienė I, Kamandulis S, Paulauskas H, Domeika A, Pliauga V, Kreivytė R, Stanislovaitienė J, Conte D. Investigating the workload, readiness and physical performance changes during intensified 3-week preparation periods in female national Under18 and Under20 basketball teams. J Sports Sci. 2020; 38(9):1018–25. 32164498 10.1080/02640414.2020.1738702

[16] Paulauskas H, Kreivyte R, Scanlan AT, Moreira A, Siupsinskas L, Conte D. Monitoring workload in elite female basketball players during the in-season phase: Weekly fluctuations and effect of playing time. Int J Sports Physiol Perform. 2019;14(7):941–8. 30676809 10.1123/ijspp.2018-0741

[17] Ferioli D, Scanlan AT, Conte D, Tibiletti E, Rampinini E. The business end of the season: A comparison between playoff and regular-season workloads in professional basketball players. Int J Sports Physiol Perform. 2021; 16(5):655–62. 33561821 10.1123/ijspp.2020-0405

[18] Foster C, Florhaug JA, Franklin J, Gottschall L, Hrovatin LA, Parker S, Doleshal P, Dodge C. A New Approach to Monitoring Exercise Training. J Strength Cond Res. 2001;15(1):109–15. 11708692

[19] Edwards S. The Heart Rate Monitor Book. Med Sci Sport Exerc. 1993; 26(5):647.

[20] Berkelmans DM, Dalbo VJ, Fox JL, Stanton R, Kean CO, Giamarelos KE, Teramoto M, Scanlan AT. The influence of different methods to determine maximum heart rate on training load outcomes in basketball players. J Strength Cond Res. 2018; 32(11):1–25. 30540282 10.1519/JSC.0000000000002291

[21] Iannaccone A, Conte D, Cortis C, Fusco A. Usefulness of Linear Mixed-Effects Models to Assess the Relationship between Objective and Subjective Internal Load in Team Sports. Int J Environ Res Public Health. 2021; 18(2):392. 33419133 10.3390/ijerph18020392 PMC7825485

[22] Nunes JA, Crewther BT, Viveiros L, De Rose D, Aoki MS. Effects of resistance training periodization on performance and salivary immune-endocrine responses of elite female basketball players. J Sports Med Phys Fitness. 2011; 51(4):676–82. 22212272

[23] Arruda AFS, Moreira A, Nunes JA, Viveiros L, De Rose D, Aoki MS, De Arruda AFS, Moreira A, Nunes JA, Viveiros L, De Rose D, Aoki MS. Monitoring stress level of Brazilian female basketball athletes during the preparation for the 2009 American CUPUP. Rev Bras Med Esporte. 2013; 19(1):44–7.

[24] Sansone P, Tessitore A, Paulauskas H, Lukonaitiene I, Tschan H, Pliauga V, Conte D. Physical and physiological demands and hormonal responses in basketball small-sided games with different tactical tasks and training regimes. J Sci Med Sport. 2019; 22(5):602–6. 30538078 10.1016/j.jsams.2018.11.017

[25] Moreira A, Arsati F, De Oliveira Lima-Arsati YB, Simões AC, De Araújo VC. Monitoring stress tolerance and occurrences of upper respiratory illness in basketball players by means of psychometric tools and salivary biomarkers. Stress Heal. 2011; 27(3):e166–72.

[26] Kamarauskas P, Conte D. The effect of basketball matches on salivary markers: a systematic review. Biol Sport. 2022;39(4):791–808. 36247952 10.5114/biolsport.2022.107481 PMC9536388

[27] Balsalobre-Fernández C, Tejero-González CM, Del Campo-Vecino J. Relationships between Training Load, Salivary Cortisol Responses and Performance during Season Training in Middle and Long Distance Runners. PLoS One. 2014; 9(8): e106066. 9(8):106066. 25153137 10.1371/journal.pone.0106066 PMC4143373

[28] Tiernan C, Lyons M, Comyns T, Nevill AM, Warrington G. Investigation of the relationship between salivary cortisol, training load, and subjective markers of recovery in elite rugby union players. Int J Sports Physiol Perform. 2020; 15(1):113–8. 10.1123/ijspp.2018-0945 31034263

[29] Springham M, Williams S, Waldron M, Mclellan C, Newton RU. Summated training and match load predictors of salivary immunoglobulin-A, alpha-amylase, testosterone, cortisol and T:C profile changes in elite-level professional football players: A longitudinal analysis. Eur J Sport Sci. 2021 Jul 16;1–11. 10.1080/17461391.2021.1949638 34181509

[30] Svilar L, Castellano J, Jukic I. Load monitoring system in top-level basketball team: Relationship between external and internal training load. Kinesiology. 2018; 50(1):25–33.

[31] Gatti R, De Palo EF. An update: Salivary hormones and physical exercise. Scand J Med Sci Sport. 2011; 21(2):157–69. 10.1111/j.1600-0838.2010.01252.x 21129038

